# Development and validation of sensitive *BCR::ABL1* fusion gene quantitation using next-generation sequencing

**DOI:** 10.1186/s12935-023-02938-2

**Published:** 2023-05-29

**Authors:** Hyeonah Lee, Jieun Seo, Saeam Shin, Seung-Tae Lee, Jong Rak Choi

**Affiliations:** 1 Department of Laboratory Medicine, Graduate School of Medical Science, Brain Korea 21 PLUS Project, Yonsei University College of Medicine, Seoul, Republic of Korea; 2 Department of Genetics, Washington University School of Medicine in Saint Louis, St. Louis, MO USA; 3 Department of Laboratory Medicine, Yonsei University College of Medicine, Seoul, Republic of Korea; 4 Dxome Co. Ltd, 8, Seongnam-daero 331beon-gil, Bundang-gu, Seongnam-si, Gyeonggi-do Republic of Korea

**Keywords:** Chronic myeloid leukemia, Fusion gene, *BCR:ABL1*, Quantification, Next-generation sequencing

## Abstract

**Background:**

*BCR::ABL1* fusion has significant prognostic value and is screened for chronic myeloid leukemia (CML) disease monitoring as a part of routine molecular testing. To overcome the limitations of the current standard real-time quantitative polymerase chain reaction (RQ-PCR), we designed and validated a next-generation sequencing (NGS)-based assay to quantify *BCR::ABL1* and *ABL1* transcript copy numbers.

**Methods:**

After PCR amplification of the target sequence, deep sequencing was performed using an Illumina Nextseq 550Dx sequencer and in-house–designed bioinformatics pipeline. The Next-generation Quantitative sequencing (NQ-seq) assay was validated for its analytical performance, including precision, linearity, and limit of detection, using serially diluted control materials. A comparison with conventional RQ-PCR was performed with 145 clinical samples from 77 patients.

**Results:**

The limit of detection of the NQ-seq was the molecular response (MR) 5.6 [*BCR::ABL1* 0.00028% international scale (IS)]. The NQ-seq exhibited excellent precision and linear range from MR 2.0 to 5.0. The IS value from the NQ-seq was highly correlated with conventional RQ-PCR.

**Conclusions:**

We conclude that the NQ-seq is an effective tool for monitoring *BCR::ABL1* transcripts in CML patients with high sensitivity and reliability. Prospective assessment of the unselected large series is required to validate the clinical impact of this NGS-based monitoring strategy.

**Supplementary Information:**

The online version contains supplementary material available at 10.1186/s12935-023-02938-2.

## Introduction

Chronic myeloid leukemia (CML) is diagnosed in approximately 1.8 per 100,000 individuals per year. About 10–15% of all adult cases of leukemia are CML[1]. CML is characterized by a *BCR::ABL1* fusion gene from the translocation between chromosomes 9 and 22 [t(9;22)]. The *BCR::ABL1* fusion gene has several pathological significance in CML patients. First, *BCR::ABL1* is a diagnostic biomarker for CML patients. The *BCR:ABL1* fusion produces a chimeric protein with constitutive tyrosine kinase activity, resulting in unregulated cell proliferation and the development of CML[2]. Second, *BCR::ABL1* is a predictive biomarker for tyrosine kinase inhibitor (TKI) eligibility. TKIs such as imatinib, nilotinib, and dasatinib selectively inhibit the growth of *BCR::ABL1* positive cells by inhibiting tyrosine kinase activity. The use of TKI dramatically improved the survival rate of patients with CML-CP[3]. Finally, the fusion gene is an efficacy biomarker for TKI response evaluation and discontinuation[4, 5].

In more than 95% of CML patients, transcripts located within e13a2 (b2a2) or e14a2 (b3a2) *BCR::ABL1* are associated with a p210 oncoprotein. Molecular testing for *BCR::ABL1* fusion is the most sensitive routine test for monitoring responses to therapy in patients with CML[5]. Time-dependent therapeutic guidelines based in part on such molecular monitoring are included in international recommendations for the management of CML[6]. Currently, real-time quantitative polymerase chain reaction (RQ-PCR) is the standard method for minimal residual disease (MRD) assessment for CML. *BCR::ABL1* transcript levels are calculated as the ratio between *BCR::ABL1* transcripts and a reference gene according to international scale (IS) values to standardize results between centers. However, RQ-PCR has several limitations, including its limit of detection, its sensitivity to PCR-inhibitors, loss of quantification precision and accuracy at low transcript concentrations, and need of a standard curve constructed from a standard material of known copy number for quantitation[7]. Therefore, alternative quantitative methods are needed to overcome the limitations of RQ-PCR.

In this study, we designed a next-generation sequencing (NGS)-based assay, Next-generation Quantitative sequencing (NQ-seq), to quantify *BCR::ABL1* and *ABL1* transcripts and validate the analytical performance of the developed assay.

## Materials and methods

### Next-generation quantitative sequencing (NQ-seq) assay procedure

For cDNA synthesis, 1 µg of total RNA was used with SuperScript IV VILO Master Mix RNA (Invitrogen, CA, USA). The cDNA (200 ng equivalent of total RNA) was amplified with primers targeting *BCR::ABL1* and *ABL1*. The PCR conditions consisted of an initial denaturation step of 95℃ for 5 min, followed by 26 cycles of 98℃ for 45 s, 60℃ for 1 min, 72℃ for 1 min, and a final elongation step at 72℃ for 5 min. The libraries were cleaned with Agencourt AMPure XP beads (Beckman Coulter, CA, USA). A total of seven sets of primers were designed for detecting the *BCR::ABL1* major types and the *ABL1* as a reference for normalization. The first target was the *BCR::ABL1* major types that covered exon 13–14 of *BCR* and exon 2 of *ABL1* (Table 1). These target primers for multiplex amplicon had the end tagged with index (p5 and p7) and universal sequencing adapter. The amplicons were clonally amplified and sequenced. Libraries were quantified using Qubit Fluorometric Quantification (Invitrogen), normalized, and processed for sequencing on a Nextseq 550Dx (Illumina, CA, USA) with a 75 bp, dual-indexed, paired-end according to the manufacturers’ instructions.

**Table 1:** Primer Sequences Used in This Study

Name	Primer Sequence 5’-3’
BCR-MAJOR-Reverse	P7-Truseq Adaptor-index-AGATGCTGACCAACTCGTG
ABL-e2-Forward 1	P5-Truseq Adaptor-index-ATGCTACTGGCCGCTGAA
ABL-e2-Forward 2	P5-Truseq Adaptor-index-TGCTACTGGCCGCTGAA
ABL-e3-REF-Reverse	P7-Truseq Adaptor-index-CTTTGAGCCTCAGGGTCTG
ABL-e3-REF-Forward 1	P5-Truseq Adaptor-index-CACCATTCCCCATTGTGAT
ABL-e3-REF-Forward 2	P5-Truseq Adaptor-index-ACACCATTCCCCATTGTGAT
ABL-e3-REF-Forward 3	P5-Truseq Adaptor-index-CACACCATTCCCCATTGTGAT

### Data processing and fusion read normalization

Raw demultiplexed NGS data were mapped to the reference genome of GRCh38 using Spliced Transcripts Alignment to a Reference (STAR) aligner tool[8]. The *BCR::ABL1* fusion junctions were called using STAR 2.7.3a, and final fusion copy numbers were determined through international scale (IS) conversion[9] with fusion and *ABL1* control reads. The test results used IS values by determining and maintaining conversion factors (CF) and molecular response (MR) to reduce variation and improve accuracy. *BCR::ABL1* transcripts types were visualized by the Arriba[10].

$${\rm{IS}}\, = \,\frac{{BCR\, - \,ABL1\,{\rm{reads}}}}{{ABL1\,{\rm{reads}}}}\,{\rm{*}}\,{\rm{CF}}$$

$${\rm{MR}}\, = \,{\rm{lo}}{{\rm{g}}_{10}}\,\left( {100\,\% \,{\rm{IS}}} \right)\, - \,{\rm{lo}}{{\rm{g}}_{10}}\,\left( {\% \,{\rm{IS}}} \right)\, = \,2\, - \,{\rm{lo}}{{\rm{g}}_{10}}\,\left( {\% \,{\rm{IS}}} \right)$$

### ***BCR::ABL1*** standard materials

We used the *BCR::ABL1* b3a2 RNA Dilution Set (Invivoscribe, San Diego, CA) to evaluate analytical performance. This standard material consists of RNA that has been extracted from confirmed *BCR::ABL1* b3a2 positive and *BCR::ABL1* negative cell lines. Also, levels not contained in this reagent (copy numbers 10^− 6^ and 10^− 7^) were manually mixed by the serial dilution method from b3a2 RNA (10^− 5^ copy numbers) positive and negative materials.

### Assay performance evaluation with positive standard materials

Precision, linearity, limit of detection (LOD), and limit of blank (LOB) were evaluated with varying levels of *BCR::ABL1* standard materials. Precision and linearity were assessed using five levels of standard materials (10^− 1^, 10^− 2^, 10^− 3^, 10^− 4^, and 10^− 5^). To evaluate assay precision, each material was measured seven times, including two or three replicates per single run on three separate days. Each dilution was measured three times to evaluate linearity. The LOD was estimated based on measurements at seven levels of dilution (10^− 1^, 10^− 2^, 10^− 3^, 10^− 4^, 10^− 5^, 10^− 6^, and 10^− 7^ dilutions) at five replicates for each. Additionally, the LOB was assessed by repeating a total of 24 times for four days using two *BCR::ABL1* negative RNA specimens consisting of subtypes b2a2 and b3a2.

### Patient samples and RNA extraction

For clinical performance validation of our assay, we used the clinical samples from CML or B-ALL patients. CML or B-ALL patients were diagnosed based on the clinical feature and test results, including complete blood count, bone marrow examination, and multiplex RT-PCR test using a HemaVision kit (DNA Technology, Aarhus, Denmark). The samples were referred to our laboratory to detect and quantify *BCR::ABL1* transcripts at diagnosis or follow-up. We selected samples with evenly distributed *BCR::ABL1* quantitative values among residual samples sufficient to perform the NGS test and compared them with the RQ-PCR test. This study was approved by the Institutional Review Board of Severance Hospital, Yonsei University Health System (4-2019-0277). Informed consent was waived for this retrospective study that evaluated anonymized samples and data and involved no potential risk to patients. Total RNA was extracted from bone marrow aspirate or whole blood with a QIAamp RNA Blood Mini Kit (QIAGEN, Hilden, Germany) according to the manufacturer’s protocol. RNA quality control and quantification were performed with an Agilent 4200 Tapestation (Agilent Technologies, CA, USA) (Table 2).

**Table 2:** Patient Characteristics

Characteristics		Patients (n = 77)^a^	
Age (Years)		55 (9–81; 43–62)	
Sex			
	Male	44 (57%)	
	Female	33 (43%)	
Diagnosis			
	CML-CP	72 (93.5%)	
	CML-AP	2 (2.6%)	
	CML-BP	1 (1.3%)	
	BLL	2 (2.6%)	
Transcript type			
	e14a2	50 (64.9%)	
	e13a2	17 (22.1%)	
	e14a2 or e13a2	10 (13.0%)	

^a^Data are median (range; IQR) or n (%)

### Real-time quantitative polymerase chain reaction (RQ-PCR)

RQ-PCR analysis for *BCR::ABL1* expression was performed using the Ipsogen kit and protocol (Ipsogen, Marseille, France). This protocol quantifies *BCR::ABL1* copy numbers relative to a total *ABL1* copy number using a real-time TaqMan method. We used 5 µL of cDNA (200 ng equivalent of total RNA) as a template in a 25 µL PCR reaction. Any *BCR::ABL1* or *ABL1* real-time PCR for individual samples was performed in duplicate.

### Statistical analysis

Statistical analysis was carried out using R version 4.02 (R Foundation for Statistical Computing, Vienna, Austria). The LOD was determined by probit analysis (95% detection rate) using the POD package, which implements the mathematical statistics presented by *Uhlig et al.*[11] in R software. The correlation was analyzed based on Pearson’s correlation coefficient, and regression analysis was performed by the least-squares method. A *p*-value < 0.05 was considered statistically significant.

## Results

### Design of ***BCR::ABL1*** NQ-seq

**Fig. 1:**
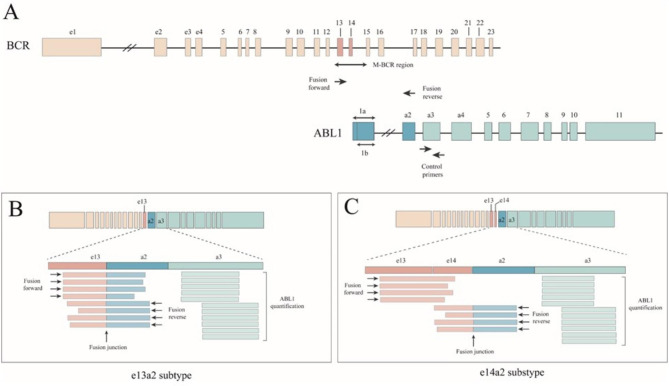
Schematic diagram of fusion differences in amplification of *BCR::ABL1* and *ABL1* reference genes. (A) *BCR::ABL1* fusion primer and *ABL1* control primer, (B) fusion copy numbers were determined to be the e13a2 subtype and *ABL1* control reads, and (C) fusion copy numbers were determined to be the e14a2 subtype and *ABL1* control reads

The break in the *BCR* gene most commonly occurs between exon 13 (e13) and exon 14 (e14) or between e14 and exon 15 (e15) in a region called the major breakpoint cluster or M-BCR[12]. The NQ-seq is designed to detect breaks in the M-BCR region, the high-frequency breakpoints of chronic CML and Philadelphia-positive acute lymphoblastic leukemia patients[13]. Two types of primer sets were designed for *BCR::ABL1* fusion copy quantification: (1) *BCR::ABL1* fusion primer and (2) *ABL1* control primer (Fig. 1A). The generated fusion amplicon has a length of either 237 bp (e13a2 subtype) or 243 bp (e14a2 subtype) spanning its fusion junction (Fig. 1B and C, and Supplementary Figure S1). Since *BCR::ABL1* fusion primer is designed in *ABL1* exon 2, *ABL1* control primers were devised in *ABL1* exon 3 for normalization of *BCR::ABL1* transcripts.

### NGS statistics

An average of 2.8 million mapped, unique reads were obtained for the total samples analyzed. The mean coverage depth of the evaluation sequencing set was an average of 18,858×, and 88.3% of the targeted bases were covered more than 200×, ensuring high fusion detection sensitivity (Supplementary Table S1).

### Assay performance evaluation with standard materials

**Fig. 2:**
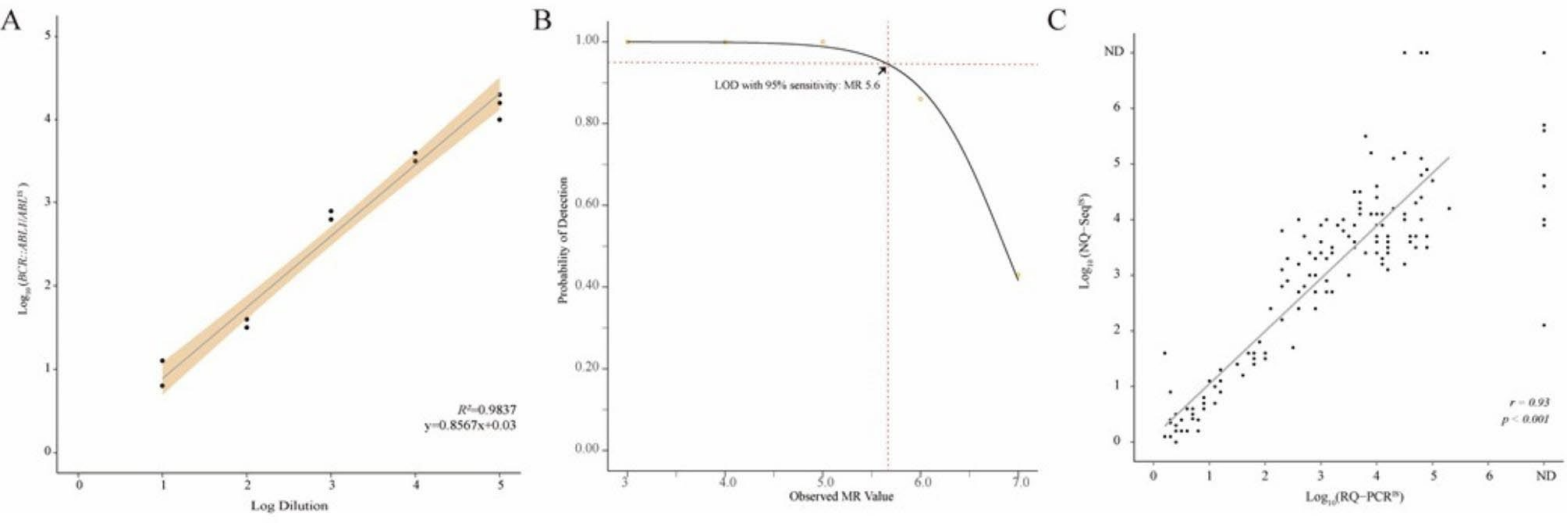
Analytical performance of the NQ-seq assay. (**A**) Linearity, (**B**) limit of detection, and (**C**) comparison with standard RQ-PCR. IS: international scale; MR: molecular response; ND: not detected; RQ-PCR: real-time quantitative polymerase chain reaction

Eight dilutions with known *BCR::ABL1* copies (10^− 1^, 10^− 2^, 10^− 3^, 10^− 4^, 10^− 5^, 10^− 6^, 10^− 7^, and negative) were tested in three batches and triplicated at each level in the final batch. The repeatability of coefficient of variation (CV) was from 3.7 to 47.2%, and the total imprecision was from 5.8 to 44.7% (Supplementary Table S2). The CVs were calculated from the MR value from the NQ-seq. Our assay exhibited an excellent correlation with expected MR values in 10^− 1^ to 10^− 5^ dilutions (*R*^*2*^ *= 0.9837, p*-value *= 5.257* × *10*^*–12*^) (Fig. 2A and Supplementary Table S3). Using probit regression, the LOD was 0.00028% IS, corresponding to MR 5.6 at 95% sensitivity (Fig. 2B). For the LOB assay, two negative reference materials were tested. Out of 25 measurements, 23 showed no *BCR::ABL1* reads, and two were positive for *BCR::ABL1* reads. The subtype b3a2 positive was found in 3 reads, and another positive read for b2a2 was acquired. Based on these results, the LOB was determined at ≤ 3 reads (0.0002–0.00056% IS) (Supplementary Table S4).

### Comparison with RQ-PCR using clinical samples

We compared NQ-seq with conventional RQ-PCR using 145 clinical samples including before and after treatment. We divided the clinical samples into three batches. The NQ-seq exhibited a good correlation with the RQ-PCR assay (*r* = 0.93, *p < 0.001*, Fig. 2C).

**Fig. 3:**
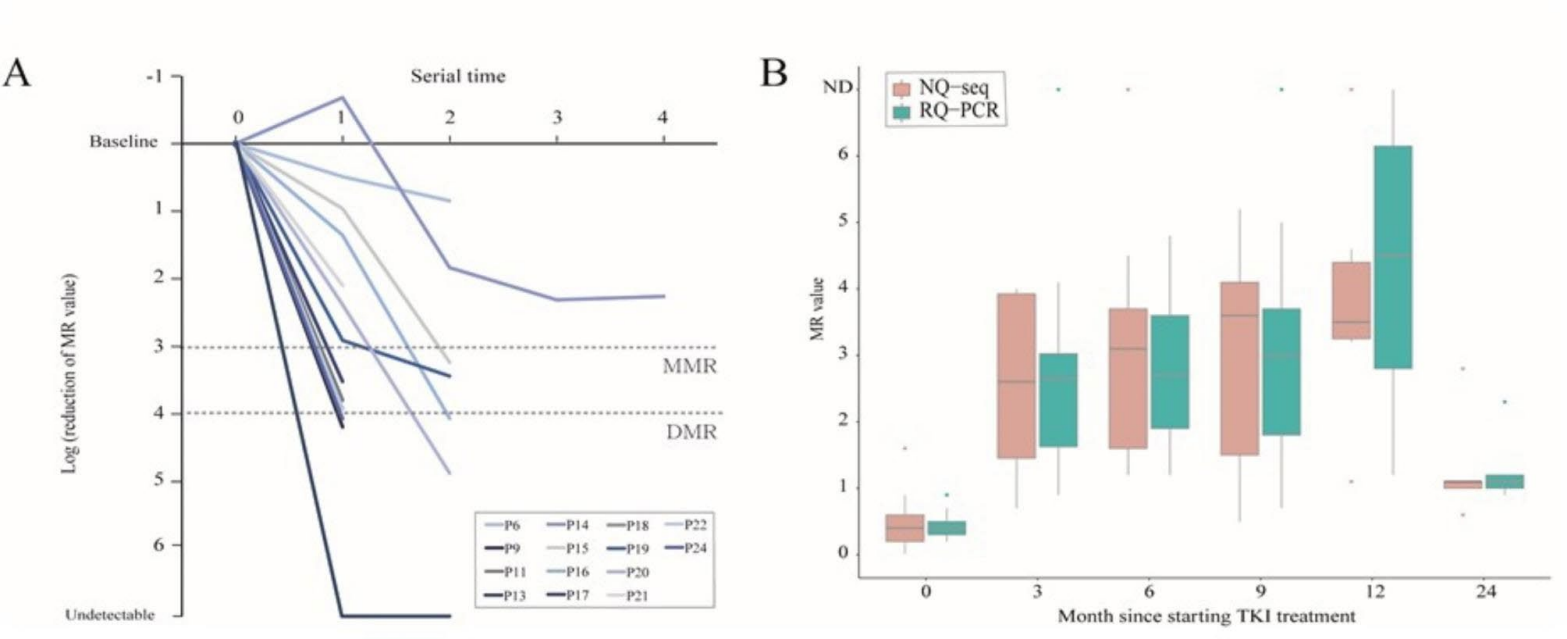
Monitoring of chronic myeloid leukemia patients during TKI treatment. (**A**) *BCR::ABL1* log reduction trend measured with NGS in samples from 37 patients who underwent follow-up tests during TKI therapy from the time of diagnosis. (**B**) Comparison of MR values of NGS and RQ-PCR according to post-treatment time points up to 12 months in serial samples. MR: molecular response; MMR: major molecular response; DMR: deep molecular response; ND: not detected; TKI: tyrosine-kinase inhibitor

Thirty-seven serial samples from 14 patients who underwent follow-up tests during TKI therapy from the time of diagnosis were quantified by NQ-seq for *BCR::ABL1* log reduction (Fig. 3A). We observed molecular responses in patients with e13a2 or e14a2. MR monitoring was assessed based on the individual baseline of 14 patients at diagnosis. Four patients achieved a deep molecular response (DMR), which we defined as ≥ 4 log reduction from baseline.

The levels of all serial samples (up to 12 months from initiation of TKI therapy) were compared with NQ-seq and RQ-PCR and displayed graphically (Fig. 3B). A total of 65 serial samples from 25 patients had no significant *BCR::ABL1* transcript level differences between quantification methods at each time point.

## Discussion

In the present study, we described the development and validation of an NGS assay to quantify the *BCR::ABL1* transcript. For monitoring and assessing molecular responses in CML patients, the current standard assay is RQ-PCR[5]. Our method demonstrated the sensitivity of MR as 5.6 (0.00028% IS), which is an excellent result compared to the sensitivity of the currently used RQ-PCR (0.0069 normalized copy number; corresponding to MR 4.2)[12]. Above all, NGS-based MRD assays do not require generation of standard curves for target quantification.

Several studies have concluded that the NGS for the fusion transcript assessment quantitative assay shows higher sensitivity than the conventional methods[14–16]. Our data corroborated superior sensitivity of 0.00028% compared to those of previous reports using targeted RNA sequencing (0.01% and 0.001%)[15, 16]. It is crucial to obtain higher precision to reduce misclassifications. The global CV of the commercial RQ-PCR kit was 25% around the MMR value (MR 3.0)[12]. The NQ-seq exhibited total imprecision (CV) of 6.0% at the MMR level and 7.2% at MR 5.0. Higher sensitivity and precision make NQ-seq useful for MRD monitoring and for selecting patients most likely to discontinue TKI treatment. However, it should be noted that not all *BCR::ABL1* reads detected by NGS may be actual reads. False-positive reads can be generated due to a PCR or sequencing error. As a result of our evaluation, the LOB of NQ-seq was determined to be three reads, so we only decided on four or more reads as positive in this study.

Moreover, this quantitative result of most patients of the analyzed cohort was obtained in concordance with RQ-PCR for quantitative assessments at the MRD level. The MR value of the patients treated with TKI by NQ-seq as log reduction in relation to individual baseline value indicated max 4.9-log reduction, showing reliable detection of deep MRs based on treatment guidelines. A 4.5-log reduction is referred to as DMR, with a higher probability of achieving treatment-free remission (TFR). TFR is achieved in CML patients who have a stable DMR without need for ongoing tyrosine kinase inhibitor treatment[17]. Meanwhile, the limitation of RQ-PCR was highlighted in long-term follow-up of CML patients applying additional MR4 and MR4.5 levels.

Several new effective MRD methods were recently considered, including droplet digital PCR (ddPCR) and NGS. According to previous studies, the ddPCR consequences suggested good performance with a confirmed LOD of MR 4.64 and a maximum CV of 9.3% with less affected primer efficiency than the conventional methods[18, 19]. We demonstrated that NGS could simultaneously determine major transcript types and MR levels with good sensitivity and acceptable precision. Moreover, NGS-based MRD assays can simultaneously perform high-throughput sequencing of multiple genes and bulk samples[20].

NQ-seq has several advantages over existing methods. Real-time quantitative PCR (RQ-PCR) and droplet digital PCR (ddPCR) are used to quantify known fusion transcript types. RQ-PCR and ddPCR can detect major *BCR::ABL1* transcripts. However, they did not differentiate between e13a2 and e14a2 types. NQ-seq can determine specific transcript types and amounts at once. This can provide additional information on sample identification, which can help improve test accuracy. In addition, a maximum of 42 samples can be performed for ddPCR and 16 samples for RQ-PCR, excluding standard material, but NQ-seq can perform 54 samples based on NextSeq 500/550 Mid Output Kit v2.5 (300 Cycles). Therefore, one can deliver more accurate and faster results to patients using NQ-seq in the clinical setting.

Future improvements to this assay include developing primer and index sets to detect atypical rare transcripts while reducing the sequencing error rate. In addition, evaluation of serial samples from large cohorts for long-term monitoring is required.

In summary, our study has developed and validated an NGS-based assay for *BCR::ABL1* quantitation that can be used in molecular monitoring in CML patients. The NQ-seq assay can be used as a reliable and promising tool for MRD monitoring in patients with CML.

## Electronic supplementary material

Below is the link to the electronic supplementary material.

Supplementary Material 1

## Data Availability

All data generated or analyzed during this study are included in this published article and its supplementary information files.

## References

[1] Rebecca L, Siegel KDM, Ahmedin Jemal DVM (2019). Cancer Statistics, 2019. CA CANCER J CLIN.

[2] Ghalesardi OK, Khosravi A, Azizi E, Ahmadi SE, Hajifathali A, Bonakchi H, Shahidi M (2021). The prognostic importance of BCR-ABL transcripts in chronic myeloid leukemia: a systematic review and meta-analysis. Leuk Res.

[3] Pophali PA, Patnaik MM (2016). The role of new tyrosine kinase inhibitors in chronic myeloid leukemia. Cancer J (Sudbury Mass).

[4] Cross NCWH, Colomer D, Ehrencrona H, Foroni L, Gottardi E, Lange T, Lion T, Polakova KM, Dulucq S, Martinelli G, Leibundgut EO, Pallisgaard N, Barbany G, Sacha T, Talmaci R, Izzo B, Saglio G, Pane F, Müller MC (2015). Hochhaus A Laboratory recommendations for scoring deep molecular responses following treatment for chronic myeloid leukemia. Leukemia.

[5] Hochhaus A, Silver MBRT, Schiffer C, Apperley JF, Cervantes F, Clark RE, Cortes JE, Deininger MW, Guilhot F, Hjorth-Hansen H, Hughes TP, Janssen JJWM, KantarjianD HM, Kim W, Larson RA, Lipton JH, Mahon FX, Mayer J, Nicolini F, Niederwieser D, Pane F, Radich JP, Rea D, Richter J, Rosti G, Rousselot P, Saglio G, Saußele S, Soverini S, Steegmann JL, Turkina A, Zaritskey A, Hehlmann R (2020). European LeukemiaNet 2020 recommendations for treating chronic myeloid leukemia. Leukemia.

[6] Michele Baccarani MWD, Rosti G, Hochhaus A, Soverini S, Apperley JF, Cervantes F, Clark RE, Jorge E, Cortes TP, Hughes HM, Kantarjian D-W, Kim RA, Larson JH, Lipton MC, Müller D, Niederwieser F, Pane JP Radich, Philippe Rousselot, Giuseppe Saglio, Susanne Saußele, Charles Schiffer, Richard Silver, Bengt Simonsson, Juan-Luis Steegmann, Goldman JM. Rüdiger Hehlmann: European LeukemiaNet recommendations for the management of chronic myeloid leukemia: 2013. *Blood* 2013, 122(6):872–884. 10.1182/blood-2013-05-501569 PMC4915804 23803709

[7] Corbisier PPL, Mazoua S, Kortekaas AM, Chung PY, Gerganova T, Roebben G, Emons H, Emslie K (2015). DNA copy number concentration measured by digital and droplet digital quantitative PCR using certified reference materials. Anal Bioanal Chem.

[8] Dobin A, Davis CA, Schlesinger F, Drenkow J, Zaleski C, Jha S, Batut P, Chaisson M, Gingeras TR (2013). STAR: ultrafast universal RNA-seq aligner. Bioinformatics.

[9] Cross NC, White HE, Ernst T, Welden L, Dietz C, Saglio G, Mahon FX, Wong CC, Zheng D, Wong S (2016). Development and evaluation of a secondary reference panel for BCR-ABL1 quantification on the International Scale. Leukemia.

[10] Uhrig S, Ellermann J, Walther T, Burkhardt P, Frohlich M, Hutter B, Toprak UH, Neumann O, Stenzinger A, Scholl C (2021). Accurate and efficient detection of gene fusions from RNA sequencing data. Genome Res.

[11] Uhlig S, Frost K, Colson B, Simon K, Mäde D, Reiting R, Gowik P, Grohmann LJA, Assurance Q. Validation of qualitative PCR methods on the basis of mathematical–statistical modelling of the probability of detection. 2015, 20:75–83.

[12] Alexis GFE, Françoise H, Carole CG, Marc B, Sandrine S, Alexandre J, Sandrine H (2020). Influence of major BCR-ABL1 transcript subtype on outcome in patients with chronic myeloid leukemia in chronic phase treated frontline with nilotinib. Oncotarget.

[13] Baccarani M, Castagnetti F, Gugliotta G, Rosti G, Soverini S, Albeer A, Pfirrmann M, International BCRABLSG (2019). The proportion of different BCR-ABL1 transcript types in chronic myeloid leukemia. An international overview. Leukemia.

[14] Sanchez R, Ayala R, Martinez-Lopez J. Minimal Residual Disease Monitoring with Next-Generation Sequencing Methodologies in Hematological Malignancies. Int J Mol Sci 2019, 20(11). 10.3390/ijms20112832 PMC6600313 31185671

[15] de Boer EN, Johansson LF, de Lange K, Bosga-Brouwer AG, van den Berg E, Sikkema-Raddatz B, van Diemen CC (2020). Detection of Fusion genes to determine minimal residual disease in Leukemia using next-generation sequencing. Clin Chem.

[16] Dillon LW, Hayati S, Roloff GW, Tunc I, Pirooznia M, Mitrofanova A, Hourigan CS (2019). Targeted RNA-sequencing for the quantification of measurable residual disease in acute myeloid leukemia. Haematologica.

[17] Pagani IS, Dang P, Saunders VA, Grose R, Shanmuganathan N, Kok CH, Carne L, Rwodzi Z, Watts S, McLean J (2020). Lineage of measurable residual disease in patients with chronic myeloid leukemia in treatment-free remission. Leukemia.

[18] Chung HJ, Hur M, Yoon S, Hwang K, Lim HS, Kim H, Moon HW, Yun YM (2020). Performance evaluation of the QXDx BCR-ABL %IS Droplet Digital PCR assay. Ann Lab Med.

[19] Bochicchio MT, Petiti J, Berchialla P, Izzo B, Giugliano E, Ottaviani E, Errichiello S, Rege-Cambrin G, Venturi C, Luciano L et al. Droplet Digital PCR for BCR-ABL1 Monitoring in Diagnostic Routine: Ready to Start? *Cancers (Basel)* 2021, 13(21). 10.3390/cancers13215470 PMC8582412 34771634

[20] Kovach AE, Raca G, Bhojwani D, Wood BL (2021). Next-generation sequencing for measurable residual Disease Assessment in Acute Leukemia. Adv Mol Pathol.

