# Supplementary material for: Development and validation of sensitive BCR::ABL1 fusion gene quantitation using next-generation sequencing
Source: Cancer Cell Int. 2023 May 29;23:106. doi: 10.1186/s12935-023-02938-2 (PMC10226238; doi:10.1186/s12935-023-02938-2)
Supplement: Supplementary file 1 — Supplementary Material 1 [file 12935_2023_2938_MOESM1_ESM.docx]

**Supplementary Table S1. NGS run summary**

| Parameter | Results |
| --- | --- |
| Uniquely mapped reads number | 2,839,398 |
| Average depth | 18,858× |
| Uniquely mapped reads% | 88.30% |

**Supplementary Table S2. Assay precision**

| NGS^IS^  (Log_10_ scale) | Repeatability (n=3) | | |  | Total imprecision (n=9) | | |
| --- | --- | --- | --- | --- | --- | --- | --- |
|  | Mean | SD | CV (%) |  | Mean | SD | CV (%) |
| 1 | 0.5 | 0.3 | 47.2 |  | 0.8 | 0.3 | 44.7 |
| 2 | 1.6 | 0.2 | 9.4 |  | 1.6 | 0.2 | 14.0 |
| 3 | 2.7 | 0.1 | 3.7 |  | 2.7 | 0.2 | 6.0 |
| 4 | 3.4 | 0.2 | 6.1 |  | 3.6 | 0.2 | 5.8 |
| 5 | 3.9 | 0.3 | 7.8 |  | 4.2 | 0.3 | 7.2 |
| MR: molecular response; SD: standard deviation; CV: coefficient of variation. | | | | | | | |
| MR=log_10_(100%IS)-log_10_(%IS) =2-log_10_(%IS) | | | | | | | |

| **Supplementary Table S3. Assay linearity evaluation**   \| Relative ratio \| Replicate \| NGS^IS^  (Log_10_ scale) \| SD \| \| CV (%) \| \| \| --- \| --- \| --- \| --- \| --- \| --- \| --- \| \| 0.1 \| 1 \| 0.8 \| 0.17 \| \| 19.25 \| \| \|  \| 2 \| 0.8 \| \|  \| 3 \| 1.1 \| \| 0.01 \| 1 \| 1.6 \| 0.06 \| \| 3.77 \| \| \|  \| 2 \| 1.5 \| \|  \| 3 \| 1.5 \| \| 0.001 \| 1 \| 2.9 \| 0.06 \| \| 2.04 \| \| \|  \| 2 \| 2.8 \| \|  \| 3 \| 2.8 \| \| 0.0001 \| 1 \| 3.6 \| 0.06 \| \| 1.62 \| \| \|  \| 2 \| 3.6 \| \|  \| 3 \| 3.5 \| \| 0.00001 \| 1 \| 4.2 \| 0.15 \| \| 3.67 \| \| \|  \| 2 \| 4.0 \| \|  \| 3 \| 4.3 \| \| MR: molecular response; SD: standard deviation; CV: coefficient of variation. \| \| \| \| \| \| \| \| MR=log10(100%IS)-log10(%IS) =2-log10(%IS) \| \| \| \|  \| \|  \| |
| --- | --- | --- | --- | --- | --- | --- | --- | --- | --- | --- | --- | --- | --- | --- | --- | --- | --- | --- | --- | --- | --- | --- | --- | --- | --- | --- | --- | --- | --- | --- | --- | --- | --- | --- | --- | --- | --- | --- | --- | --- | --- | --- | --- | --- | --- | --- | --- | --- | --- | --- | --- | --- | --- | --- | --- | --- | --- | --- | --- | --- | --- | --- | --- | --- | --- | --- | --- | --- | --- | --- | --- | --- | --- | --- | --- | --- | --- | --- | --- | --- | --- | --- | --- | --- | --- | --- |

| **Supplementary Table S4. Limit of blank evaluation** | | | | | | | | | | | | | | |
| --- | --- | --- | --- | --- | --- | --- | --- | --- | --- | --- | --- | --- | --- | --- |
| Sample | IS Level (%) | Day 1 | | Day2 | | Day3 | | | | Day4 | | | | Observed proportion (%) |
|  |  | #1 | #2 | #1 | #2 | #1 | #2 | #3 | #4 | #1 | #2 | #3 | #4 |  |
| N1 | Negative | ND | ND | ND | ND | ND | ND | ND | ND | 0.00056 | ND | ND | ND | 91.7 |
| N2 |  | ND | ND | ND | ND | 0.0002 | ND | ND | ND | ND | ND | ND | ND |  |
| ND: Not detected | | | | | | | | | | | | | | |

**Supplementary Figure S1.** Detection of *BCR-ABL1* transcript type visualized by Arriba. (A) The representative case of e13a2 transcript type: Patient 14 and (B) The representative case of e14a2 transcript type: Patient 9.

**
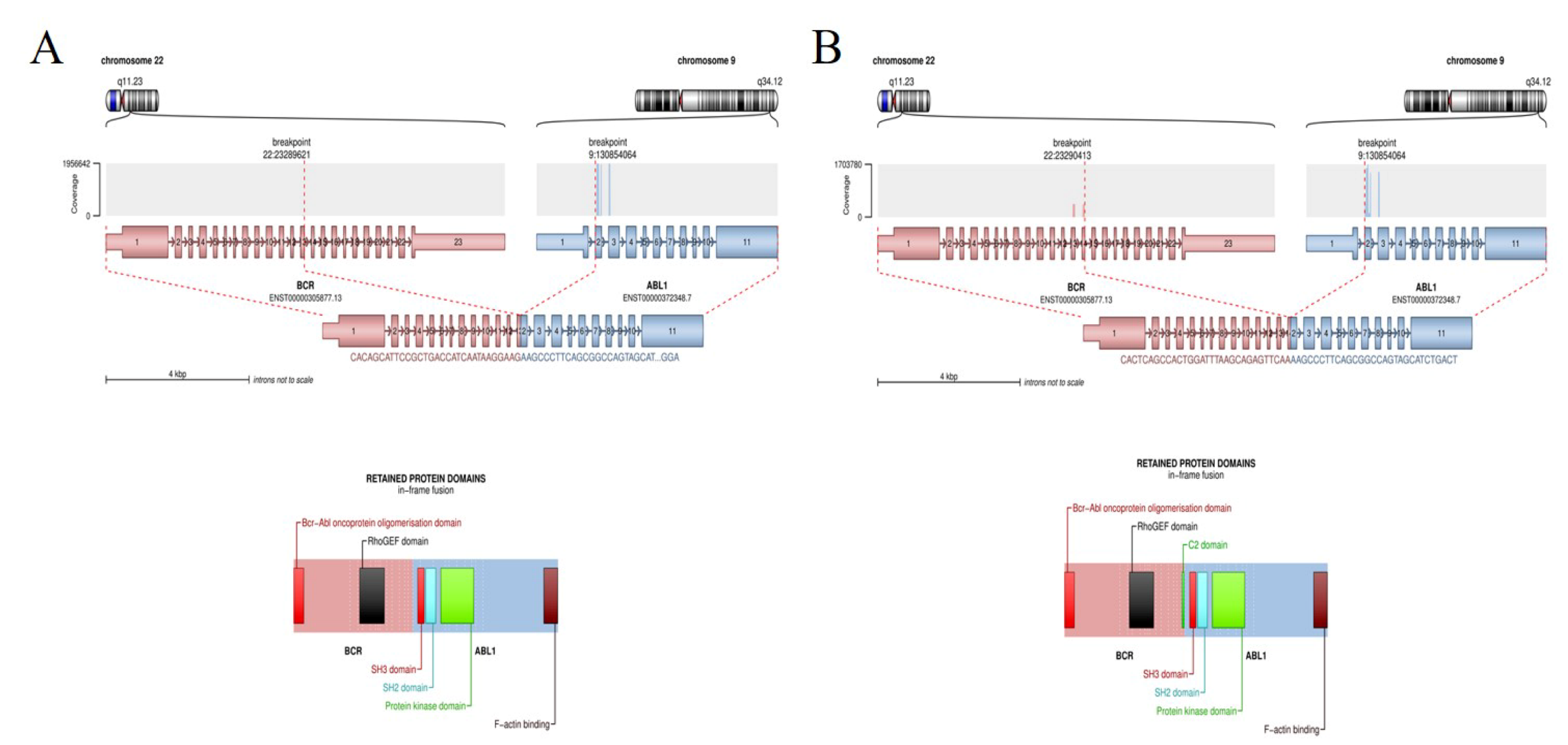
**
